# Vanishing Bone Metastases in Superior Vena Cava Obstruction

**DOI:** 10.5334/jbsr.2927

**Published:** 2022-11-07

**Authors:** Mimoun Mimoun Mazlin, Wim Verwimp, Stephane Dechambre

**Affiliations:** 1 Centre Hospitalier de Mouscron, BE

**Keywords:** vanishing bone metastases, Intravertebral venous collateral

## Abstract

**Teaching Point:** Intravertebral venous collateral formation can occur in thoracic venous obstruction syndrome and mimic metastatic bone lesions on contrast-enhanced imaging: vanishing bone metastases.

## Introduction

Intravertebral venous collateral formation can occur in thoracic venous obstruction syndrome and mimic metastatic bone lesions on contrast-enhanced imaging.

## Case History

A 60-year-old man was admitted to the emergency department complaining of headaches, dyspnea and deterioration of consciousness during the past few days. He has a history of esophageal cancer treated with surgery and adjuvant radiochemotherapy. Clinical examination revealed swelling of the face, neck and upper limbs, as well as turgidity of the jugular veins and stridor.

**Figure 1:**
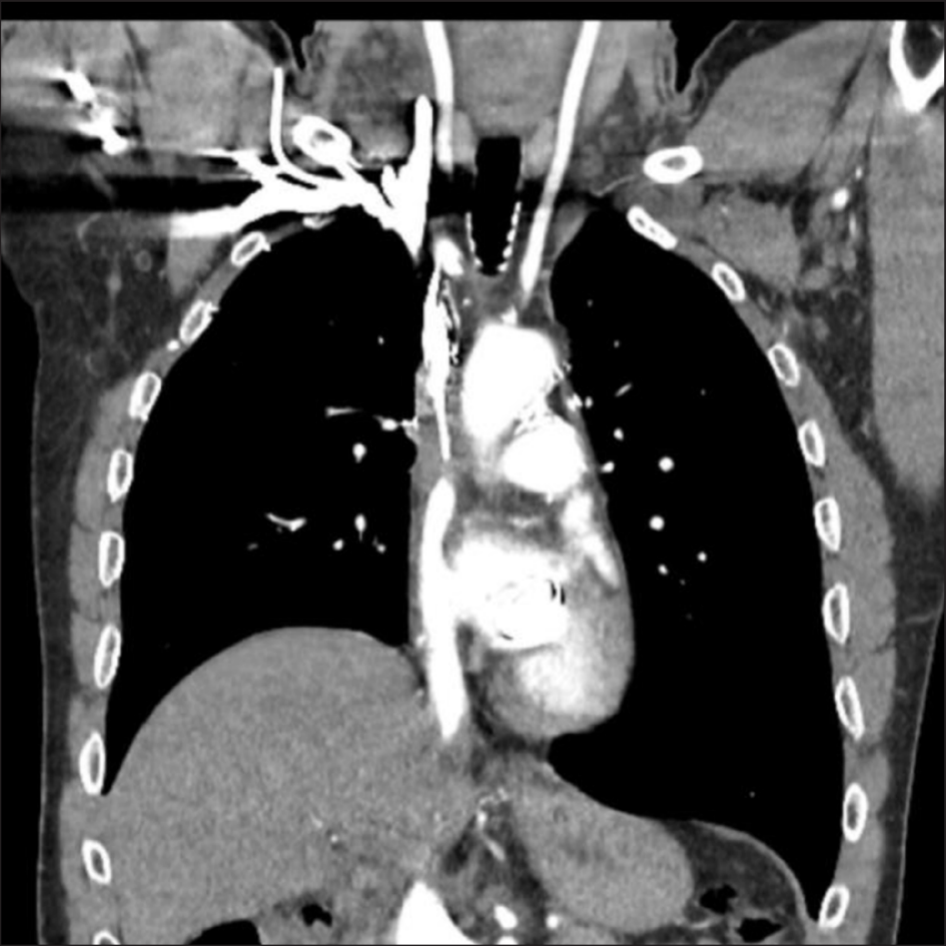


**Figure 2:**
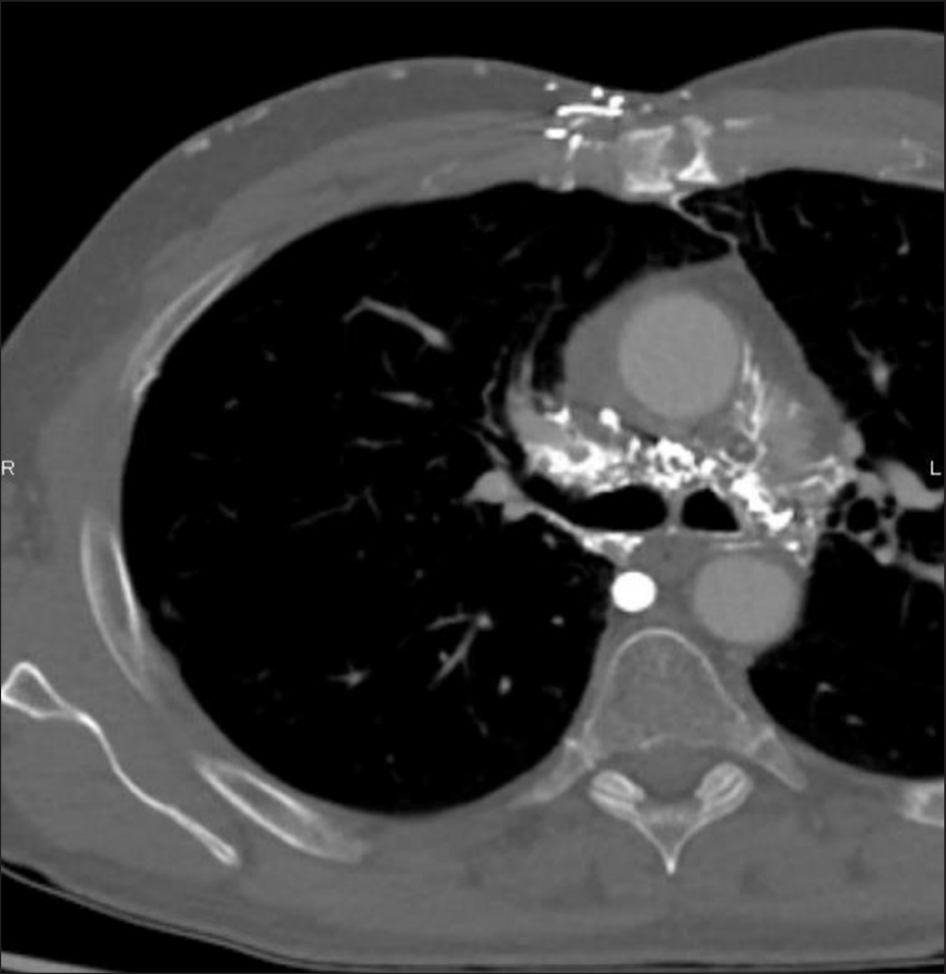


A contrast enhanced computed tomography (CT) scan of the neck and chest was performed. It showed a superior vena cava obstruction with various venous collaterals (Figures 1 and 2) [1].

**Figure 3:**
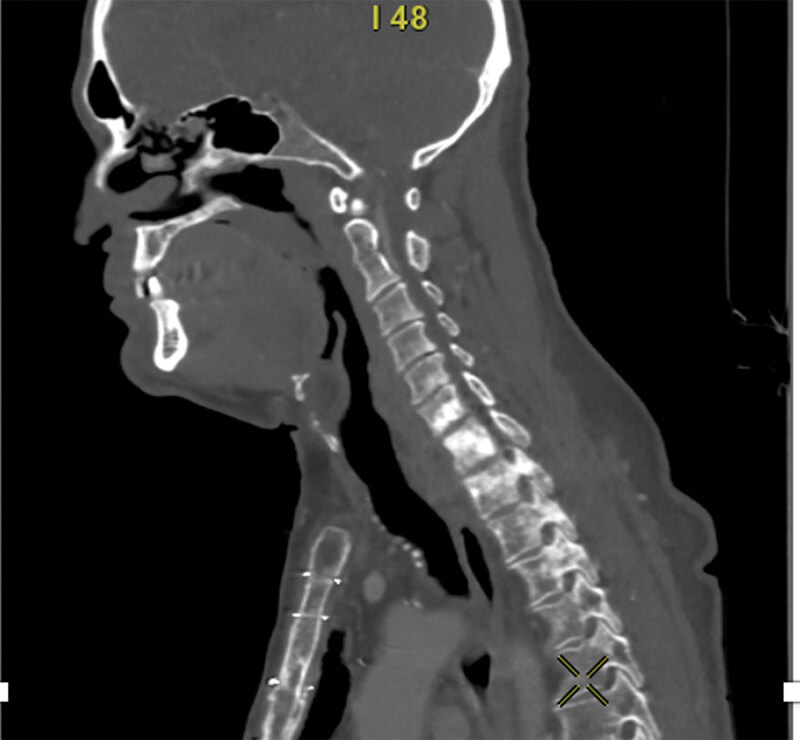


**Figure 4:**
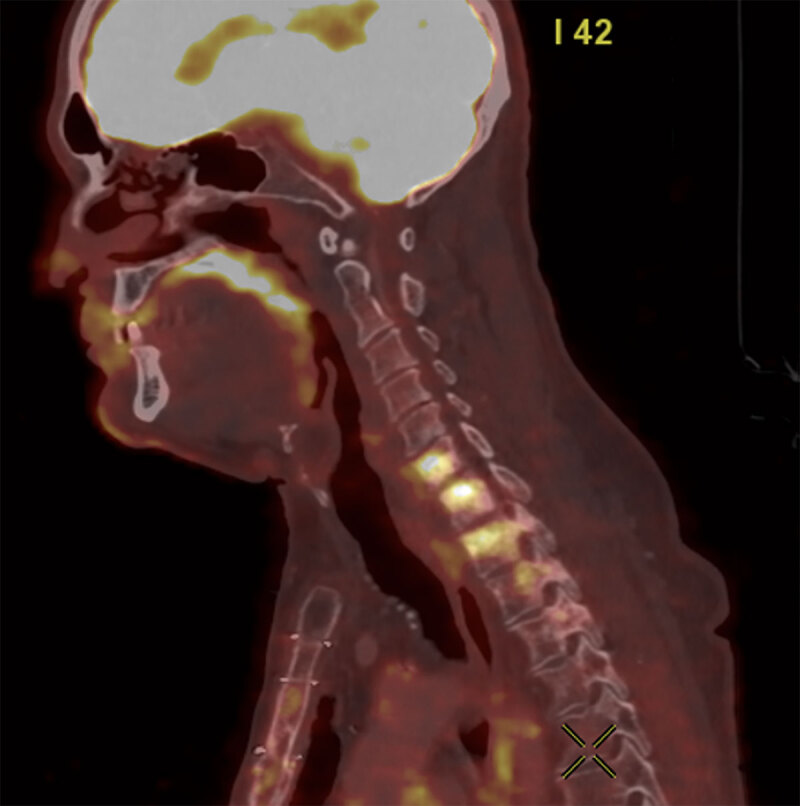


Multiple high-density bone lesions were also detected in several cervical and thoracic vertebral bodies (Figure 3). These findings were not present on a previous CT scan performed two months earlier. Initially these lesions were suggested to be compatible with rapidly progressive bone metastases. A FDG PET-CT showed that these lesions were hypermetabolic (Figure 4).

**Figure 5:**
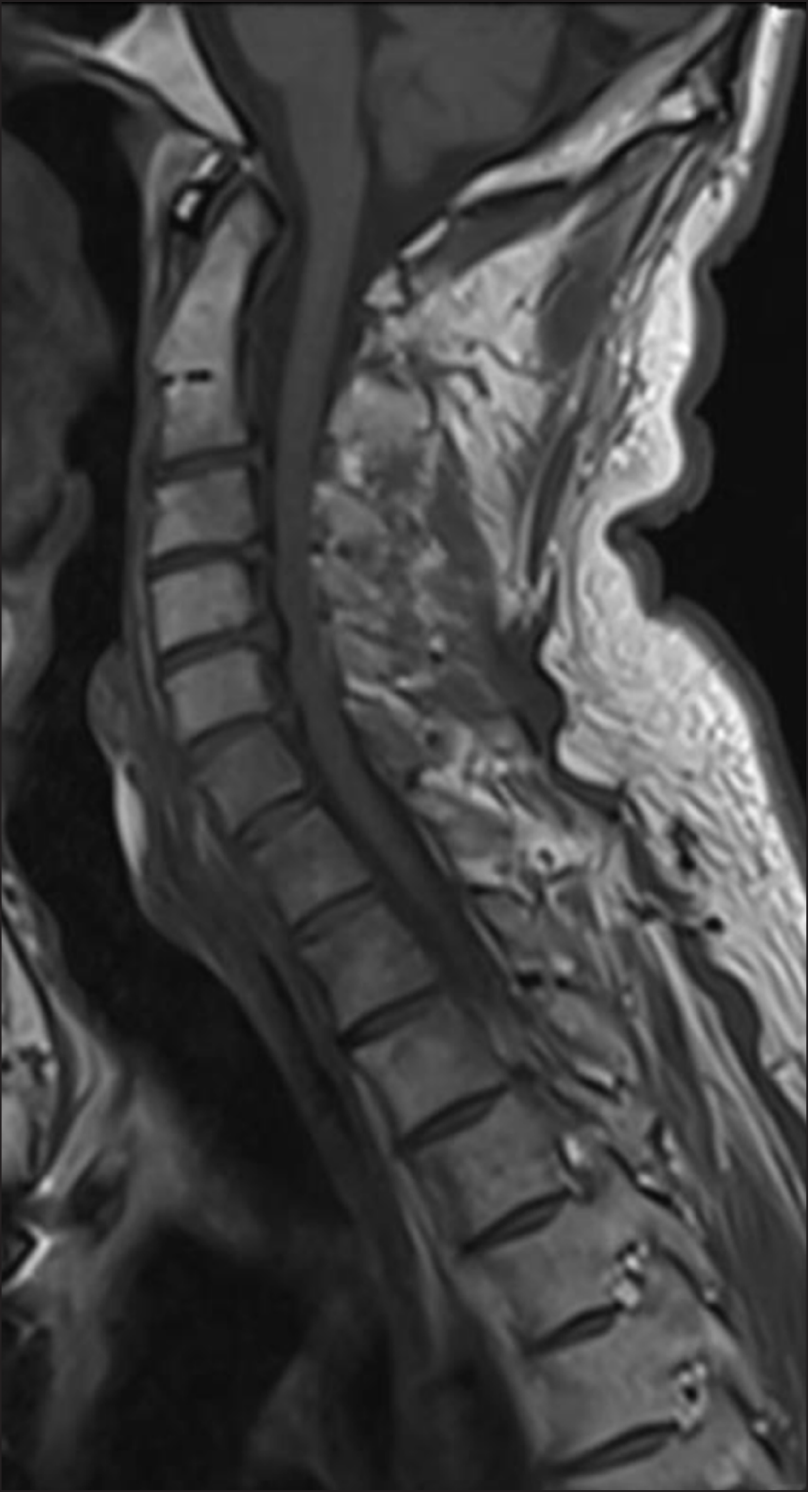


**Figure 6:**
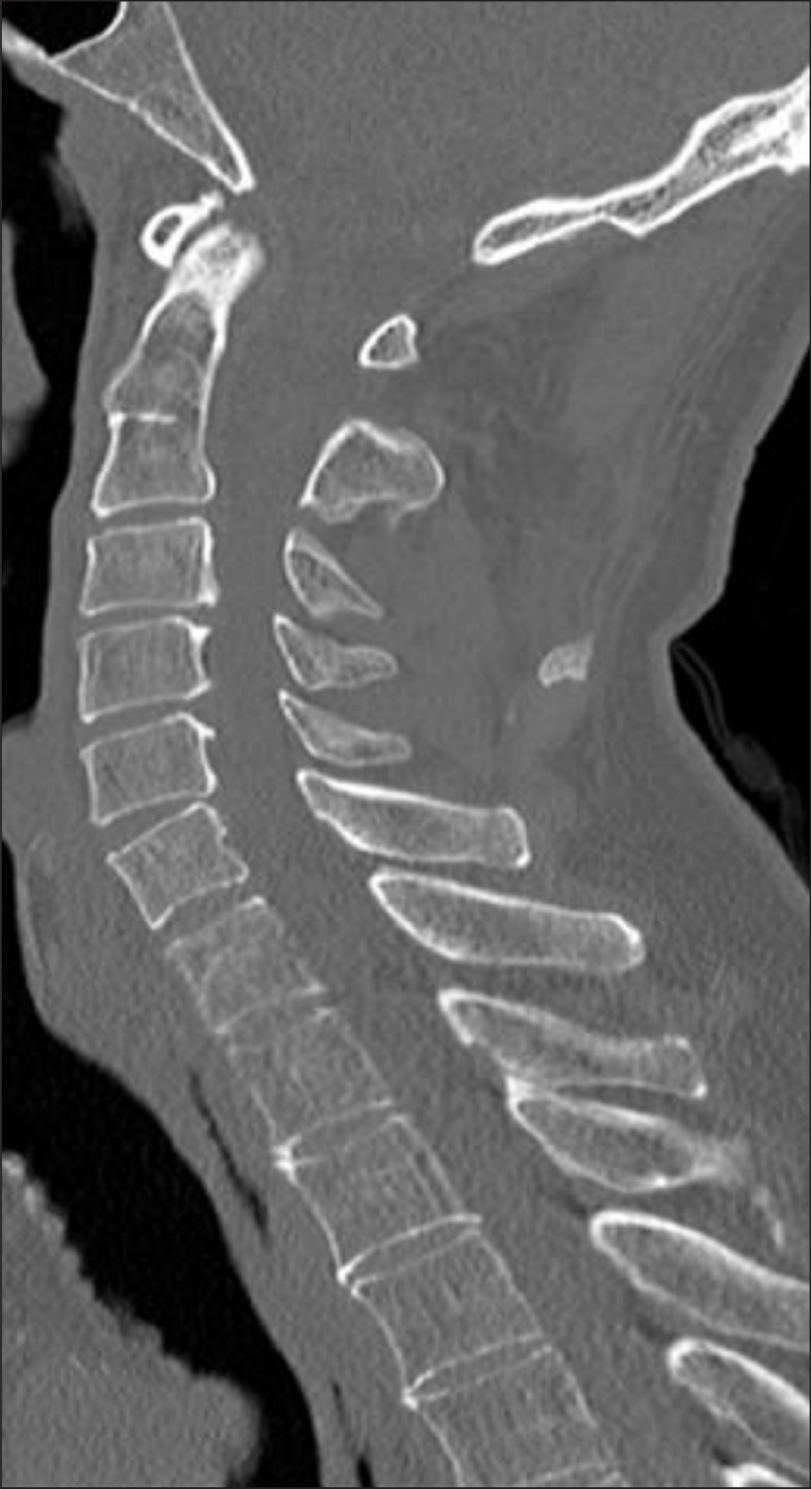


Spine magnetic resonance imaging (MRI) performed two days later didn’t reveal any vertebral signal abnormalities (Figure 5). Nor were there any bone condensing metastatic lesions depicted on a non-enhanced CT scan of the cervical spine performed after the MRI (Figure 6).

Therefore, we can conclude that the dense intravertebral images on the contrast-enhanced CT imaging resulted well from contrast accumulation in intravertebral venous collaterals [2].

## Discussion

Venous dilatation and relative blood stagnation can be present when the superior vena cava is obstructed. Most frequent venous collateral pathways are via the azygos, hemiazygos, intercostal veins and vertebral venous plexuses. In some cases, intravertebral collaterals can also be involved. These intraosseous venous collaterals are located in the vertebral bodies and drain into vertically oriented veins in the spinal subarachnoid space [3,4,5].

Therefore, stagnation of intravenous contrast material in these intravertebral collaterals can mimic condensing bone metastases on CT scans after contrast administration [6], hence they are called vanishing bone metastases.

In literature, there are limited number of case reports describing such vanishing bone metastases. One of the first descriptions of this peculiar aspect was in 2009 by Jesinger and colleagues [2]. It was further illustrated in 2021 by Fukamizu and his team [7].

## Conclusion

Vanishing metastases appear to be an underdescribed entity and should not be confused for malignant lesions.

## References

[1] Sheth S, Ebert MD, Fishman EK. Superior vena cava obstruction evaluation with MDCT. Am J Roentgenol. 2010; 194: W336–W346. DOI: 10.2214/AJR.09.2894 20308479

[2] Jesinger RA, Huynh B, Gover D. Superior vena cava syndrome resulting in osseous venous congestion simulating sclerotic bone lesions. AJR Am J Roentgenol. 2009; 192: W344–W345. DOI: 10.2214/AJR.08.2068 19457801

[3] Eckenhoff JE. The vertebral venous plexus. Can J Anaesth. 1971; 18: 487–95. DOI: 10.1007/BF03026011 5094099

[4] Holemans JA, Howlett DC, Rankin SC. Case report: Superior vena cava obstruction: unusual CT findings due to venous collaterals. Clin Radiol. 1997; 52: 559–60. DOI: 10.1016/S0009-9260(97)80337-6 9240713

[5] Kapur S, Paik E, Rezaei A, Doan NV. Where there is blood, there is a way: Unusual collateral vessels in superior and inferior vena cava obstruction. Radiographics. 2010; 30: 67–78. DOI: 10.1148/rg.301095724 20083586

[6] Thomas N, Oliver TB, Sudarshan T. Vanishing bone metastases–A pitfall in the interpretation of contrast enhanced CT in patients with superior vena cava obstruction. Br J Radiol. 2011; 84: e176–e178. DOI: 10.1259/bjr/50676625 21849358 PMC3473785

[7] Fukamizu EMN, Seabra A, Otto DY, Sawamura MVY, Bordalo-Rodrigues M, Helito PVP. Vanishing bone metastasis: Pictorial essay. Radiol Bras. 2021; 54(5): 336–340. DOI: 10.1590/0100-3984.2020.0124 34602670 PMC8475168

